# Health economic evaluation of newborn hepatitis B immunization prevention strategies in Ningbo: a Markov modeling study

**DOI:** 10.3389/fpubh.2025.1532604

**Published:** 2025-04-16

**Authors:** Wei Feng, Zhengxiong Li, Mingkuan Fan, Sijia Yang, Yuqi Shao, Kui Liu, Shuaishuai Huang, Sanjun Fu

**Affiliations:** ^1^Fenghua District Center for Disease Control and Prevention, Ningbo, China; ^2^School of Medical Informatics and Engineering, Xuzhou Medical University, Xuzhou, China; ^3^School of Medicine, Xiangyang Polytechnic, Xiangyang, China; ^4^Ningbo Municipal Center for Disease Control and Prevention, Ningbo, China; ^5^Zhejiang Provincial Center for Disease Control and Prevention, Hangzhou, China; ^6^Ningbo Yinzhou No.2 Hospital, Ningbo, China

**Keywords:** hepatitis B, vaccination, mother-to-child transmission, economic evaluation, Markov model

## Abstract

**Background:**

Hepatitis B virus (HBV) infection poses a significant public health challenge in China. The Prevention of mother-to-child Transmission (PMTCT) strategy of combining universal hepatitis B vaccination with hepatitis B immunoglobulin (HBIG) for newborns is crucial in preventing widespread infection. In this study, we conduct health economic evaluation of three strategies: PMTCT, universal vaccination, and non-vaccination for newborns in Ningbo, China.

**Methods:**

This study developed a decision-Markov model and simulated a cohort of 100,000 newborns to assess the cost-effectiveness and cost–benefit of three strategies from a healthcare system perspective. The primary outputs included total costs, life-years (LYs), quality-adjusted life-years (QALYs), incremental cost-effectiveness ratios (ICERs), benefit–cost ratios (BCRs). One-way and probabilistic sensitivity analyses (PSA) were performed to verify the robustness of the model.

**Results:**

Among the three strategies, the PMTCT results in the least disease burden and mortality related to hepatitis B. In comparison to a cohort of 100,000 unvaccinated infants, the PMTCT is expected to prevent 6,029 cases of acute symptomatic infections, 27,348 HBV carriers, 4,170 chronic infections, 3,597 cases of cirrhosis, 2,911 cases of hepatocellular carcinoma (HCC), and 3,930 HBV-related deaths. The ICERs for PMTCT and universal vaccination were − 56,371.77 yuan/QALY and − 56,654.77 yuan/QALY, respectively. The BCRs for PMTCT and universal vaccination were 19.13 and 15.95, respectively, when compared to no vaccination. The PSA revealed that all ICER scatter points are situated within the fourth quadrant, and the probability of PMTCT being cost-effective exceeds 90%.

**Conclusion:**

Implementing universal hepatitis B vaccination with HBIG for newborns in Ningbo demonstrated high cost-effectiveness, making the continuation of the PMTCT strategy highly recommended.

## 1 Introduction

Hepatitis B virus (HBV) infection poses a significant global public health challenge, often resulting in chronic infection, cirrhosis, and hepatocellular carcinoma (HCC) (1–3). In 2019, approximately 316 million individuals were afflicted with chronic HBV infection, resulting in 555,000 fatalities and 1.82 million disability-adjusted life years associated with this disease (4, 5). China accounts for a disproportionately high share, with one-third of the global hepatitis B surface antigen (HBsAg) carrier population and 55% of the annual liver cancer deaths linked to HBV (6). Mother-to-child transmission is the primary route of HBV spread (7, 8). Newborns infected with HBV are more likely to develop chronic hepatitis B (CHB), cirrhosis, or HCC (9). Therefore, preventing mother-to-child transmission (PMTCT) of HBV is crucial for reducing the burden of hepatitis B.

Neonatal HBV vaccination has been identified as a cost-effective strategy to address this public health issue (10–12). China has made remarkable progress in implementing HBV vaccination. Since 1992, vaccination has been recommended for all newborns nationwide and was integrated into the Expanded Program of Immunization in 2002. In 2011, China launched the PMTCT program, which includes HBsAg screening for pregnant women and provision of hepatitis B vaccine (HepB) combined with hepatitis B immunoglobulin (HBIG) for infants born to HBsAg-positive mothers (13, 14). Over three decades, these efforts have resulted in significant declines in child HBsAg seroprevalence, from 9.67% in 1992 to 0.32% in 2014 for children aged 1–4 years, and from 10.74 to 0.90% for children aged 5–14 years (15, 16).

The reliability of the economic evaluation of HBV prevention strategies critically depends on the model used. The Markov model, which simulates the natural progression of HBV infection in a population and includes a comprehensive range of economic evaluation metrics, has been widely adopted for such evaluations. Cost–benefit analysis (CBA) and cost-effectiveness analysis (CEA) are useful for evaluating the economic and health benefits of HBV prevention strategies. When conducting an economic analysis using a Markov model, it is essential to consider parameters such as the prevalence of HBV infection, mortality rates, and healthcare costs associated with HBV-related diseases. Given that these parameters often vary by region, it is necessary to explore these findings in a specific area. Ningbo, as a representative of China’s economically developed eastern coast region, has maintained over 98% coverage for neonatal HBV vaccinations since 1989. However, a comprehensive economic evaluation of the HBV immunization strategies in this region has not yet been reported. Therefore, this study aimed to evaluate the economic benefits of PMTCT and universal hepatitis B vaccination versus no vaccination for newborns in Ningbo, providing valuable insights for health policymakers to optimize HBV prevention strategies.

## 2 Materials and methods

### 2.1 Comparator strategies

In this study, we compared three scenarios: PMTCT, universal vaccination, and a hypothetical scenario of non-vaccination. The universal vaccination strategy involves 3-dose hepatitis B vaccination series (HepB3), each containing 10 μg. The initial dose was administered within 24 h of birth, followed by the second and third doses at one and six months of age, respectively. The PMTCT strategy included HBsAg-screening for pregnant women. Newborns of HBsAg-positive mothers receive an additional HBIG within 24 h of birth, along with the universal vaccination strategy. Newborns of HBsAg-negative mothers followed the universal vaccination strategy and did not receive HBIG. No vaccination is a scenario without any interventions.

### 2.2 Model overview

**Figure 1:**
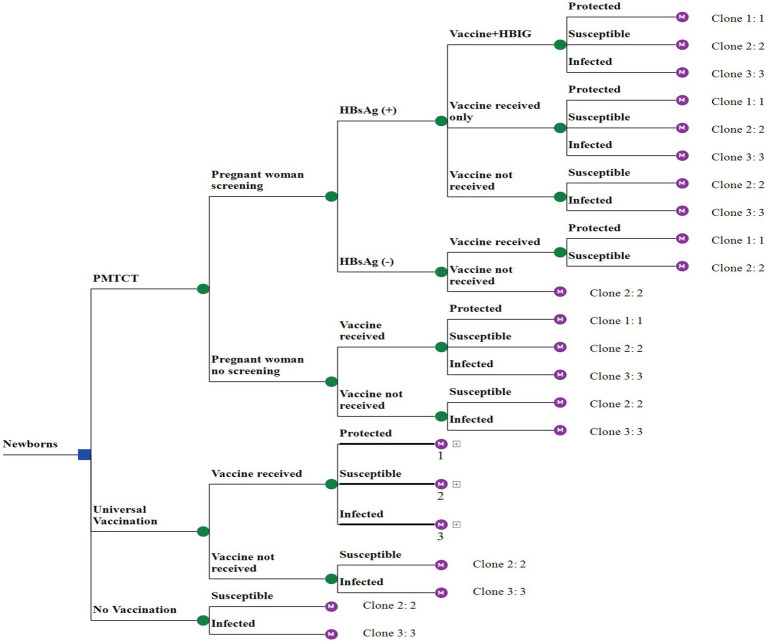
Decision tree model structure for the intervention comparison.

A decision tree-Markov model was used to illustrate the Markov processes of vaccine protection, HBV-susceptible, and HBV-infected (Figure 1). The HBV-susceptible involved nine health states: susceptible, acute hepatitis B (AHB), HBsAg seroclearance, HBV carriers, CHB, compensated cirrhosis (CC), decompensated cirrhosis (DC), HCC, and death Supplementary File 1 (markov model of vaccine protection and HBV-susceptible), where AHB is a temporary state in the susceptible state, not an independent health state. The HBV-infected process excluded the states of susceptible and AHB, while the remaining states were consistent with the HBV-susceptible process. The model was constructed using TreeAge Pro 2021 (TreeAge Software, Inc., Williamstown, MA, USA). To simulate the model, we assumed a cohort of 100,000 individuals for a maximum of 80 cycles corresponding to life expectancy, with each cycle representing 1 year.

### 2.3 Epidemiological and vaccination parameters

According to monitoring data from the Ningbo Center for Disease Control and Prevention, the vaccination rate for newborns who received three doses of 10 μg of the HBV vaccine was 98.29%, with an estimated range of 96.60 to 99.75%. The HBsAg screening rate among pregnant women was 99.0%, and the HBsAg-positivity rate among the screened pregnant women was 7.92%. Among infants born to HBsAg-positive mothers, 91.3% received HBIG. These data were acquired from a survey conducted in Ningbo (17). For infants born to HBsAg-positive mothers who received both HBIG and the HepB3 vaccine, the vaccine efficacy was 93.0% compared to 87.0% for those who received only the HepB3 vaccine (18–20). The perinatal infection rate for infants receiving both HBIG and HepB3 vaccines was 4.9%, while it was 8.0% for those receiving only the HepB3 vaccine (21, 22). All vaccinated newborns were assumed to receive the full process of HepB3, and the protective effects of the HBV vaccine were considered lifelong, without taking into account the effects of herd immunity resulting from hepatitis B vaccination. Additional details of the relevant parameters are presented in Table 1.

**Table 1:** Model parameters inputs and the ranges of the sensitivity analysis.

Parameters	Base-case value	Range	Distribution	Reference
Coverage (%)				
HBsAg-screening rate	99.00	98.00 ~ 100.00	Triangular	(17)
Coverage rate of HepB3	98.29	96.60 ~ 99.75	Triangular	(17)
Coverage rate of HepB3 plus HBIG	91.30	87.98 ~ 94.62	Triangular	(17)
Efficacy (%)				
HBsAg+ mothers: HepB3+ HBIG	93.00	88.90 ~ 94.70	Triangular	(18–20)
HBsAg+ mothers: HepB3	87.00	82.20 ~ 90.70	Triangular	(18–20)
HBsAg-mothers: HepB3	97.66	96.60 ~ 98.73	Triangular	(17)
General newborns: HepB3	96.67	95.52 ~ 97.82	Triangular	(17)
Epidemiology data (%)				
Prevalence of HBsAg+ mothers	7.94	7.06 ~ 8.28	Triangular	(17)
Prenatal infection rate (%)				
HBsAg+ mothers: HepB3+ HBIG	4.87	3.58 ~ 6.59	Triangular	(21)
HBsAg+ mothers: HepB3	8.00	5.70 ~ 9.30	Triangular	(18, 22)
HBsAg+ mothers: Unvaccination	40.34	32.28 ~ 48.40	Triangular	(23, 24)
General newborns: HepB3	0.93	0.41 ~ 1.82	Triangular	(25)
General newborns: Unvaccination	3.22	2.58 ~ 3.87	Triangular	(25)
Vaccination-related costs (CNY)				
HepB3	36.76	±25%	Gamma	(17)
HBIG (per dose)	209.85	±25%	Gamma	(17)
HBsAg-screening	22.09	±25%	Gamma	(17)
Direct medical costs of HBV-related (CNY)				
No hospitalization for AHB	871.37	±25%	Gamma	(17)
Hospitalization for AHB	19,593.15	±25%	Gamma	(17)
Fulminant hepatitis B	117,122.20	±25%	Gamma	(17)
HBV carriers	821.54	±25%	Gamma	(26)
CHB	26,784.81	±25%	Gamma	(17)
CC	71,095.32	±25%	Gamma	(17)
DC	102,992.65	±25%	Gamma	(17)
HCC	152,608.55	±25%	Gamma	(17)
Health utility				
General population	0.82	0.80 ~ 0.84	Beta	(18, 21)
AHB	0.76	0.66 ~ 0.79	Beta	(18, 21)
Fulminant	0.26	0.15 ~ 0.35	Beta	(18, 21)
HBV carriers	0.79	0.76 ~ 0.82	Beta	(27)
CHB	0.77	0.76 ~ 0.87	Beta	(28, 29)
CC	0.72	0.66 ~ 0.75	Beta	(18, 21)
DC	0.57	0.47 ~ 0.61	Beta	(18, 21)
HCC	0.51	0.39 ~ 0.57	Beta	(18, 21)
Discount rate (%)	3	0 ~ 6	Fix	(30)

HepB3, 3-dose hepatitis B vaccination series; HBsAg, hepatitis B surface antigen; AHB, acute hepatitis B; CHB, chronic hepatitis B; CC, compensated cirrhosis; DC, decompensated cirrhosis; HCC, hepatocellular carcinoma.

### 2.4 Cost and health utility

The cost analysis in this study was conducted from a healthcare system perspective, encompassing both the costs of the vaccination program and direct medical costs associated with HBV-related diseases. The cost of the HepB3, maternal HBsAg-screening and HBIG was determined from a field survey conducted in Ningbo (17). Direct medical costs included outpatient, hospitalization, and self-purchased drug expenses. Data on the costs associated with HBV-related diseases were primarily obtained from surveys conducted in Ningbo City and Zhejiang Province (17, 25). All costs were inflated to reflect the 2022 price level according to the healthcare consumer price index from the Ningbo Statistical Yearbook.

Health utility is a numerical measure that evaluates the quality of life and typically ranges from 0 to 1. In this study, utility values were mainly derived from studies conducted in China that assessed various types of HBV-related utilities (18, 21, 27–29). Furthermore, the cost and utility values were discounted at a rate of 3% (30). All costs and utilities are presented in Table 1.

### 2.5 Transition probability

In this study, the transition probabilities within the Markov model were obtained from published literature (31–44) (Table 2). In the susceptible Markov model, newborns were assumed to begin in a susceptible state, with possible transitions to other states. The probability of becoming carriers due to perinatal HBV infection was estimated to be 0.885 (36). Therefore, for the perinatal infection Markov model, we set the initial probability of HBV carriers and HBsAg seroclearance to 0.89 and 0.11, respectively. In addition, the age-specific mortality rate in Ningbo was used to define the probability of death from other causes in the general population.

**Table 2:** Transition probabilities used in the Markov model.

State transition	Base-case value	Range	Reference
From susceptible to AHB	(b1 + b2*exp.(b3*(age+1)))*(1-pFOIdecrease)^(19 + age)		(31, 32)
	b1 = 0.034435	0.023542 ~ 0.045328	
	b2 = 0.494480	0.371654 ~ 0.617305	
	b3 = −0.729443	−0.942076 ~ −0.516809	
	pFOIdecrease = 0.02162	0.02162 ~ 0.03243	
From AHB to symptomatic	0.05 Age < 20; 0.2 Age ≥ 20	0 ~ 0.5	(31)
From symptomatic to hospitalization	0.3	0.1 ~ 0.5	(33)
From hospitalization			
To fulminant hepatitis B	0.05 Age < 20; 0.04 Age ≥ 20	0 ~ 0.1	(33)
To death	0.6 Age < 20; 0.7 Age ≥ 20	0.04 ~ 0.8	(34, 35)
From AHB to HBV carriers	0.89 Age < 1; exp.(−0.65*(age^0.46)) Age ≥ 1	±50%	(36)
From HBV carriers			
To HBsAg seroclearance	0.012 Age < 20; 0.0226 Age ≥ 20	±50%	(37, 38)
To CHB	0.0012 Age < 20; 0.0023 20 ≤ Age<40; 0.0054 Age ≥ 40	±50%	(39)
To HCC	0.002	0 ~ 0.0021	(40)
From CHB			
To HBV carriers	0.00608	0 ~ 0.033	(41)
To CC	0.015 Age < 20; 0.02 20 ≤ Age<40; 0.027 Age ≥ 40	±50%	(42)
To HCC	0.005	0 ~ 0.005	(43)
From CC			
To DC	0.073	0.035 ~ 0.1	(44)
To HCC	0.0316	0.0258 ~ 0.0374	
From DC			
To HCC	0.0316	0.0258 ~ 0.0374	(44)
To death	0.17	0.1 ~ 0.25	
From HCC to death	0.34	0.22 ~ 0.45	

### 2.6 Economic analysis

We simultaneously performed CEA and CBA based on the decision-analytic model, and calculated the total costs, life-years (LYs), quality-adjusted life-years (QALYs), incremental cost-effectiveness ratios (ICERs) and benefit–cost ratios (BCRs) of implementing PMTCT and universal vaccination strategies with non-vaccination as the reference scenario. According to WHO recommendations, a strategy is considered cost-effective if the ICER is ≤3 times the gross domestic product (GDP) per capita and highly cost-effective if the ICER does not exceed 1 GDP per capita (30). The GDP per capita in Ningbo for 2022 (CNY 163,911) was adopted as the WTP threshold. If the BCR > 1 indicate a positive benefit and a higher BCR signifies greater benefits of the immunization program (45).

### 2.7 Sensitivity analyses

To assess the robustness of model results, we conducted both one-way and probabilistic sensitivity analyses (PSA). In the one-way sensitivity analysis (OWSA), parameters were varied within a range of ±25% (or ± 50%) from their baseline values to evaluate their impact. Other parameter ranges used in this analysis are presented in Table 1, and the results are illustrated using a tornado diagram. For the PSA, we performed second-order Monte Carlo simulations with 1,000 iterations, utilizing the pre-specified distributions for all parameters. The results are depicted in an ICER scatter plot and a cost-effectiveness acceptability curve (CEAC).

## 3 Results

### 3.1 Base-case analysis

We estimated the health outcomes of HBV infection for each strategy in a simulated cohort of 100,000 newborns (Table 3), with PMTCT resulting in the fewest HBV-related diseases and deaths. Compared with no vaccination, the PMTCT strategy in a cohort of 100,000 newborns would prevent 6,029 cases of acute symptomatic infection, 27,348 HBV carriers, 4,170 chronic infections, 3,597 cases of cirrhosis, 2,911 cases of HCC, and 3,930 HBV-related deaths. The base-case results were shown in Table 4. And the PMTCT strategy incurred the lowest total cost at CNY 1,407.99 and achieved the highest LYs and QALYs at 29.64 and 24.30, respectively. Additionally, the BCR for PMTCT was 19.13, which was higher than that of universal vaccination (15.95).

**Table 3:** Health outcome cases in a simulated birth cohort under three scenarios.

Strategy	Symptomatic AHB	HBV carriers	CHB	Cirrhosis	HCC	HBV-related death
PMTCT	258	1,427	189	73	106	168
Universal vaccination	275	1,887	256	144	170	251
No vaccination	6,287	28,775	4,359	3,670	3,017	4,098
PMTCT vs. No vaccination	−6,029	−27,348	−4,170	−3,597	−2,911	−3,930
Universal vaccination vs. No vaccination	−6,012	−26,888	−4,103	−3,526	−2,847	−3,847

**Table 4:** Base-case analysis results of two immunization strategies compared to no vaccination.

Strategy	Cost (CNY)			LYs	QALYs	ICER (¥/QALY)	BCR
	Immune cost	Illness cost	Total cost				
No vaccination	–	28,263.69	28,263.69	29.26	23.82	–	–
Universal vaccination	36.13	1,633.03	1,669.16	29.63	24.29	−56,654.77	15.95
PMTCT	73.06	1,334.93	1,407.99	29.64	24.30	−56,371.77	19.13

LYs, life-years; ICER, incremental cost-effectiveness ratio; QALYs, quality-adjusted life-years; BCR, benefit–cost ratio. CNY: Chinese yuan (¥).

### 3.2 One-way sensitivity analysis

**Figure 2:**
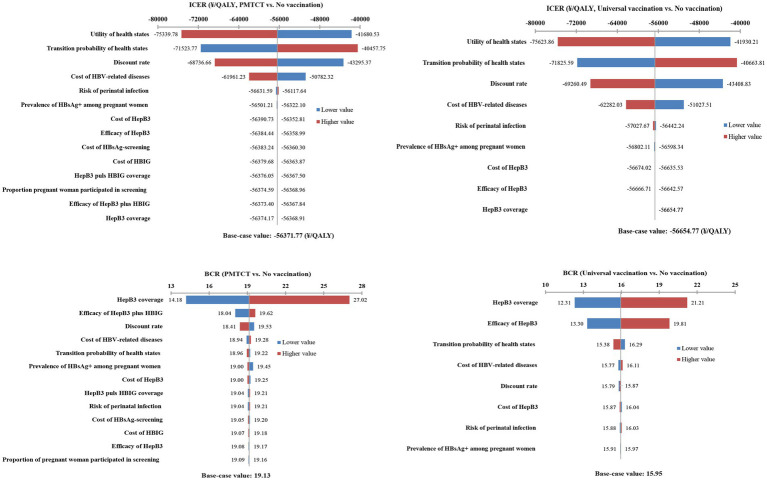
Tornado diagram of the one-way sensitivity analysis.

In the CEA, the tornado diagram demonstrated that the health utility, transition probabilities, discount rate, and cost of HBV-related diseases were the top four critical parameters influencing the ICER. The ICER remained consistently negative when all parameters fluctuated within their ranges. In the CBA, the greater impact on BCR was on HepB3 coverage, efficacy of HepB3 plus HBIG, and HepB3 vaccine efficiency. However, within the range of variation of these parameters, the BCR remained greater than 1. Consequently, the model was very stable regardless of key parameter changes (Figure 2).

### 3.3 Probabilistic sensitivity analysis

**Figure 3:**
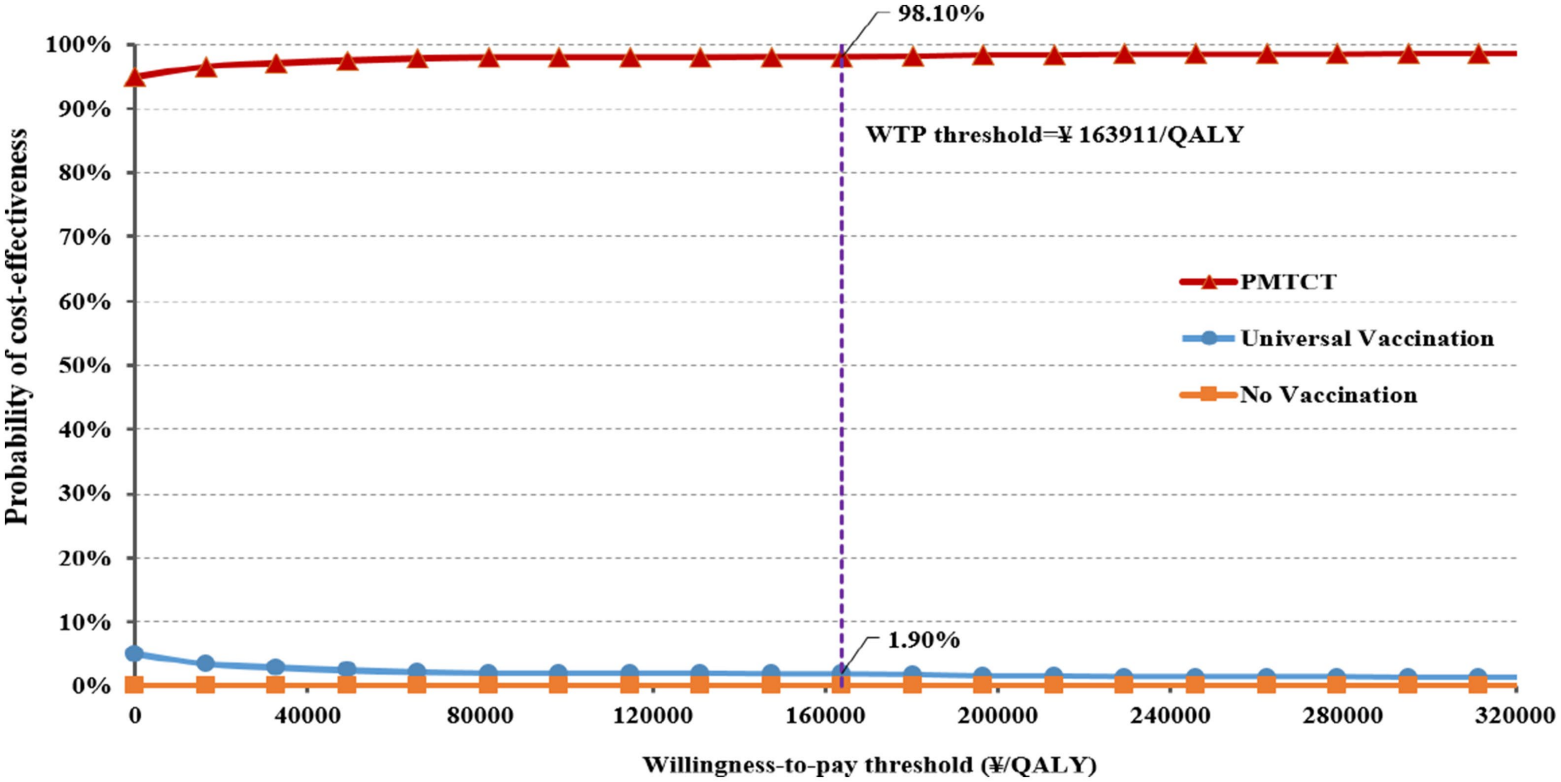
Cost-effectiveness acceptability curves for three alternative scenarios.

The results of the PSA for the incremental cost-effectiveness scatter plot are shown in Supplementary File 2 (Incremental cost-effectiveness scatter plot). All scatter points in the plot are located in the fourth quadrant of the incremental cost-effectiveness plane, indicating the two immunization strategies are superior to no vaccination. Furthermore, the CEAC results demonstrate that the probability of PMTCT being more cost-effective remains over 90% as the WTP increases (Figure 3). At the WTP threshold of ¥163,911/QALY, the probability that the PMTCT strategy is cost-effective is 98.10%, making it more cost-effective than no vaccination and universal vaccination.

## 4 Discussion

Mother-to-child transmission is the primary route of HBV transmission, accounting for 40–50% of new HBV infections (46, 47). In China, approximately 6% of pregnant women are HBV carriers (48, 49), and 90% of infants are at risk of developing CHB after infection (8, 50). Implementing a PMTCT strategy is crucial for preventing HBV transmission and reducing the burden of HBV infection. Compared to universal vaccination, the PMTCT strategy has proven to be a more effective preventive intervention (25, 51, 52). A study conducted in Taiwan also found that the PMTCT strategy was more cost-effective than universal vaccination, and this conclusion was not affected by the HBsAg positivity rate (53). In this study, we used both CEA and CBA evaluation methods to show that PMTCT strategy can achieve greater economic benefits and fewer HBV-related diseases and deaths compared to universal vaccination and non-vaccination, making it a more cost-effective strategy that deserves continued promotion.

Although the PMTCT strategy is more cost-effective, some underdeveloped areas in China have low HBsAg-screening rates among pregnant women, with rates as low as 49.2% (13). This suggests that, while promoting the PMTCT strategy, we should not overlook the universal vaccination. Our study found that the cost-savings per QALY gained from the universal vaccination program were higher than those from the PMTCT, indicating that universal vaccination is also a highly cost-effective measure. Economic evaluations in Italy (54), Vietnam (55), and Iran (56) have also highlighted the cost-effectiveness of universal HepB vaccination for infants, making it an economically efficient preventive strategy. Another reason for not neglecting the universal vaccination strategy is the low timely HepB vaccination rate in the remote western regions of China. In 2015, the timely HepB vaccination rate in western China was only 90.80%, with 35.46% of the counties having a timely vaccination rate below 90% (13). In these regions, the universal vaccination program was easier to implement compared to the PMTCT strategy.

The economic evaluation of hepatitis B plays a crucial role in the control and elimination of this disease. Economic evaluations related to global hepatitis B elimination indicate that neonatal universal vaccination has successfully averted 210 million cases of CHB worldwide as of 2015. If the global objective of eliminating hepatitis B is realized, approximately 7.3 million hepatitis B-related deaths could be prevented between 2015 and 2030 (57). Reliable economic evaluations empower policymakers to identify optimal strategies for hepatitis B prevention, thereby strengthening their commitment to disease elimination. The reliability of economic evaluation is critically dependent on the economic models employed. Currently, the decision tree-Markov model is widely used on the economic evaluations of hepatitis B prevention (25, 51, 52, 56). This study also utilizes the decision tree-Markov model, and sensitivity analyses confirm the stability of the economic evaluation of implementing the PMTCT program for newborns. Under the different WTP, acceptable probability of cost-effectiveness of the PMTCT consistently greater than 90%. Similar to studies conducted in China and Namibia, implementing a PMTCT strategy for neonates provides more cost-effective compared to no vaccination (25, 52).

However, this study has some limitations. First, while antiviral prophylaxis for pregnant women with high HBV-DNA levels can reduce the likelihood of perinatal HBV transmission (46, 58), we did not include maternal antiviral prophylaxis in our model. Further research should consider antiviral prophylaxis for pregnant women. Secondly, although we endeavored to incorporate local data from Ningbo City into our model, there may still be discrepancies between our model’s parameters and real-world conditions. These differences could potentially affect the generalizability of our findings. Finally, the Markov model used in this study is a static model that is inadequate to fully simulate the dynamic transmission of HBV across a wider population.

## 5 Conclusion

The current PMTCT program in Ningbo is highly cost-effective compared to non-vaccination, significantly saving costs and reducing the disease burden associated with HBV-related ailments. Given the economic and health benefits of this strategy, China’s health policymakers should sustain this initiative and allocate continuous financial resources for its continued implementation.

## Data Availability

The original contributions presented in the study are included in the article/Supplementary material, further inquiries can be directed to the corresponding authors.
