# Supplementary material for: Health economic evaluation of newborn hepatitis B immunization prevention strategies in Ningbo: a Markov modeling study
Source: Front Public Health. 2025 Apr 16;13:1532604. doi: 10.3389/fpubh.2025.1532604 (PMC12040845; doi:10.3389/fpubh.2025.1532604)
Supplement: Supplementary file 1 [file Data_Sheet_1.docx]

Supplementary Material

# Supplementary Figures


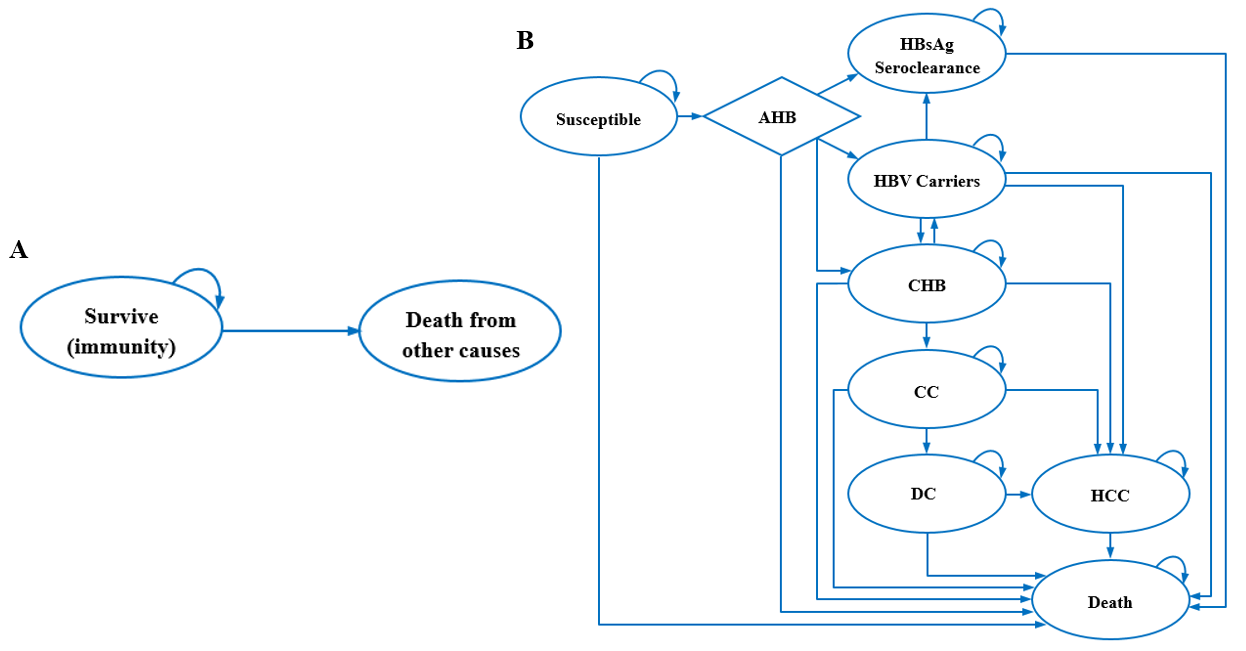


Figure 2 Markov model of vaccine protection (A) and HBV-susceptible (B). HBsAg, hepatitis B surface antigen; AHB, acute hepatitis B; CHB, chronic hepatitis B; CC, compensated cirrhosis; DC, decompensated cirrhosis; HCC, hepatocellular carcinoma.
