# Supplementary material for: Health economic evaluation of newborn hepatitis B immunization prevention strategies in Ningbo: a Markov modeling study
Source: Front Public Health. 2025 Apr 16;13:1532604. doi: 10.3389/fpubh.2025.1532604 (PMC12040845; doi:10.3389/fpubh.2025.1532604)
Supplement: Supplementary file 2 [file Data_Sheet_2.docx]

Supplementary Material

# Supplementary Figures

**
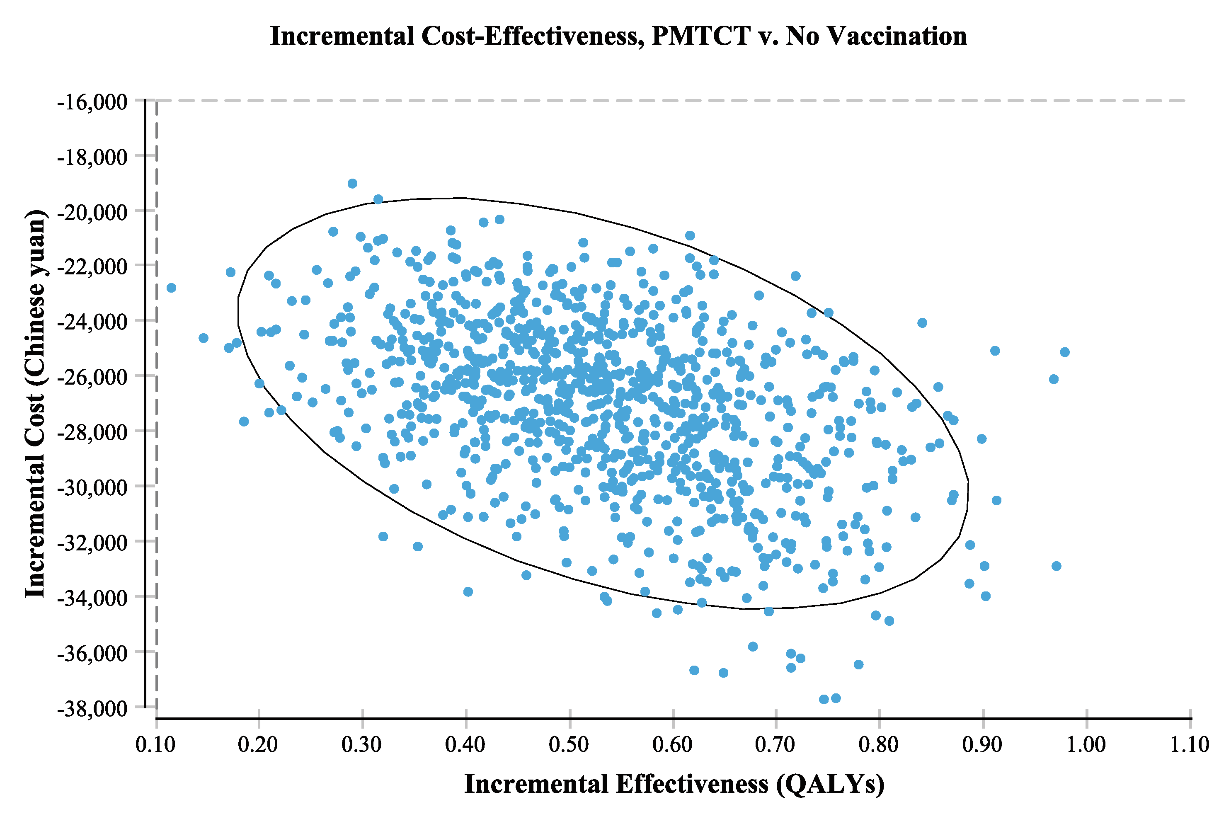
**

Figure 1 Incremental cost-effectiveness scatter plot for the PMTCT strategy compared to no vaccination.


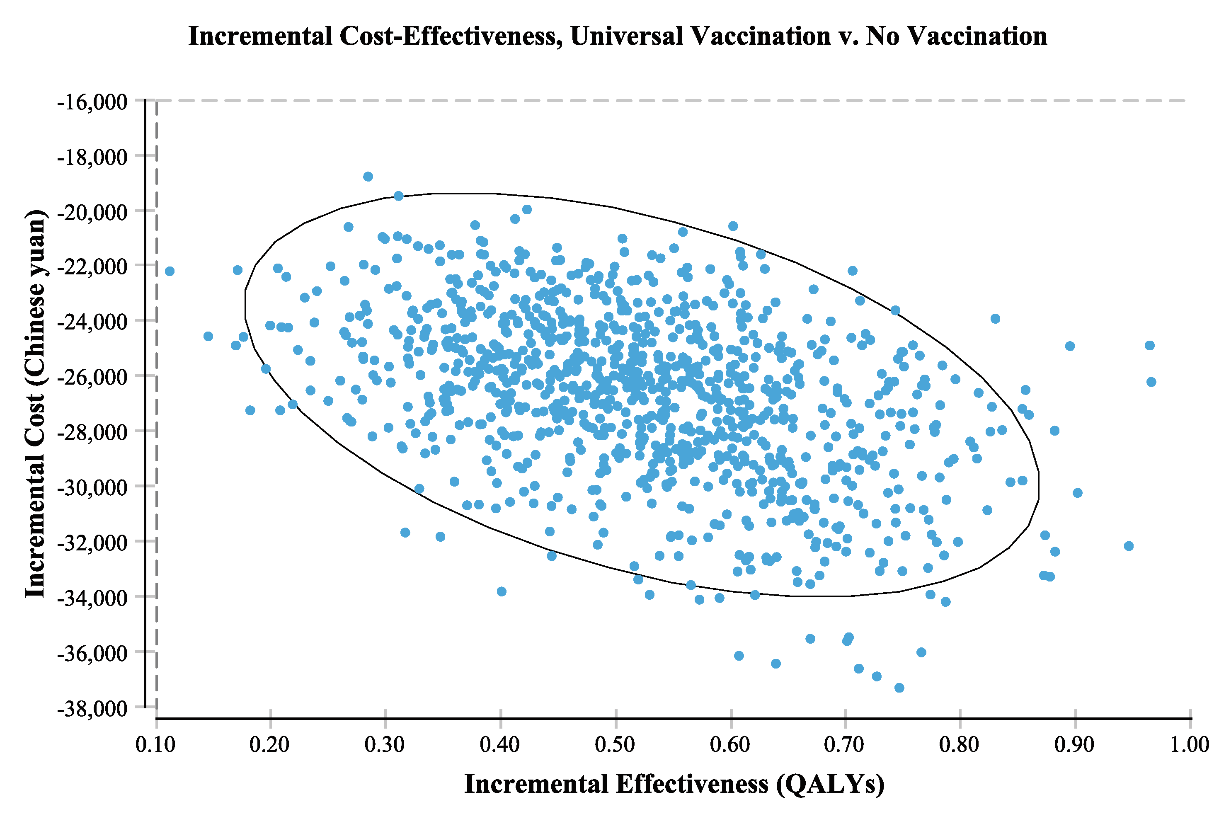


Figure 2 Incremental cost-effectiveness scatter plot for the universal vaccination strategy compared to no vaccination
